# The comparison of albumin and 6% hydroxyethyl starches (130/0.4) in cardiac surgery: a meta-analysis of randomized controlled clinical trials

**DOI:** 10.1186/s12893-021-01340-x

**Published:** 2021-09-11

**Authors:** Ling Wei, Dongping Li, Lin Sun

**Affiliations:** 1 Department of Rehabilitation, The Second Xiangya Hospital, Central South University, Changsha, 410011 China; 2 Department of Cardiothoracic Surgery, The Second Xiangya Hospital, Central South University, Changsha, China; 3 Hunan Key Laboratory of Kidney Disease and Blood Purification, Department of Nephrology, Second Xiangya Hospital, Central South University, Changsha, 410011 China

**Keywords:** Albumin, Hydroxyethyl starches, Cardiac surgery, Randomized controlled clinical trials, Meta-analysis

## Abstract

**Background:**

Fluid administration is a key tool in the maintenance of normovolemia in patients with cardiac surgery. The trials that evaluated the safety of 6% hydroxyethyl starch (HES) 130/0.4 in cardiac surgical patients were inconsistent. It is necessary to compare the efficacy and safety of albumin and 6% HES (130/0.4).

**Method:**

We searched for the randomized controlled clinical trials that compared human albumin with 6% HES (130/0.4) in cardiac surgery in PubMed, Cochrane, and Embase.

**Results:**

Ten studies involved a total of 1567 patients were included in our meta-analysis. For the efficiency, there was no difference in total volume of infusion between compared groups [P = 0.64, Fixed Effect Model (FEM): standardized mean difference (SMD) = 0.04, 95% confidence interval (CI) (− 0.12, 0.20)]. As for safety, the albumin show more risk than hydroxyethyl starch 130/0.4 in blood loss [P = 0.02, FEM: SMD: 0.22, 95% CI (0.03, 0.41)]. There was no difference in the frequency of transfusions (P = 0.20, RR = 1.11; 95% CI (0.95, 1.27)) between the two groups. No difference was observed for the days in intensive care unit [P = 0.05, FEM: SMD = − 0.18, 95% CI (− 0.36, 0.00)], and the days in hospital [P = 0.32, FEM: SMD = − 0.11, 95% CI (− 0.32, 0.10)]. Furthermore, both the incidence of AKI, RRT, and mortality were comparable in the two groups.

**Conclusion:**

This study provided evidence that the 6% HES (130/0.4) might be the substitute for HA, which reduced the economic burden for patients with cardiac surgery. However, the effect of 6% HES (130/0.4) and HA on AKI still needs to be further studied.

**Supplementary Information:**

The online version contains supplementary material available at 10.1186/s12893-021-01340-x.

## Background

Fluid administration is a crucial tool in the maintenance of normovolemia in patients with cardiac surgery. There is a higher requirement for hemodynamic stabilization in cardiac surgery. The management of fluid administration is essential for preventing postoperative acute kidney injury (AKI) and mortality in cardiac surgery patients [1, 2]. Furthermore, cardiopulmonary bypass (CPB) in cardiac surgery activates the clotting system and increases the damage to the coagulation function [3]. The selection of fluids during the priming of CPB and perioperative volume expansion might influence the risk of excessive clotting. The crystalloids, albumin, and synthetic colloids, such as hydroxyethyl starch (HES), were commonly used as administered fluids [4]

The common choice for restrictive fluid therapy in cardiac surgery was albumin, and synthetic colloids, owing to their effective volume expansion effect compared to crystalloids. Human albumin (HA) has a negligible impact on blood clotting, but it carries high costs and risks of infection. Both albumin and synthetic colloids might provoke allergic reactions [5]. HA is commonly used in cardiac surgery because it can coat the fluid pathway surface, prevent platelet consumption and reduce inflammatory mediators [6, 7]. Furthermore, HA can maintain a stable colloid oncotic pressure [8]. The third-generation hydroxyethyl starch (HES), such as 6% HES (130/0.4), has been developed [9]. 6% HES (130/0.4) has lower molar substitution and molecular weight, which results in rapid clearance and metabolism [10]. More importantly, they were less costly than albumin. Therefore, HES 130/0.4 was considered a substitute for human albumin (HA) and crystalloids in cardiac surgery. However, trials that evaluated the safety of 6% HES (130/0.4) in cardiac surgical patients were inconsistent.

Thus, it is necessary to compare the safety and efficacy of albumin and new generation hydroxyethyl starch (HES). We conducted a systematic meta-analysis by searching randomized controlled studies that compare albumin and HES130/0.4 in cardiac surgery. The outcomes included total volume infusion, blood loss, transfusion requirements, the incidence of AKI and RRT, ICU and hospital stays, and mortality.

## Methods

We searched for the randomized controlled clinical trials in PubMed, Cochrane, and Embase by the end of June 9th, 2020. The included studies compared human albumin with hydroxyethyl starches (130/0.4) in cardiac surgery. The retrieval strategy was shown in Additional file 1. We checked related systematic reviews to avoid missing suitable studies. Eligible publications were not restricted with time. The ethics committee’s approval was waived because this manuscript was based on the data of published articles. Therefore, informed consent is also not required.

### Study selection

Inclusion criteria: (1) randomized controlled clinical trials (RCT); (2) the patients had cardiac surgery; (3) studies that compared albumin to hydroxyethyl starches (130/0.4). Two independent authors screened the possible articles by reading the title or abstract. If there were any disagreements, the final decision for whether to include the study was made based on the full text of the publications and discussion between the reviewers.

### Data collection

Two reviewers independently collected all data from included articles according to the predetermined data collection sheets. If there were any discrepancies, a third reviewer participated in discussions and solutions. We collected the safety and efficacy data for two fluids. The outcome of safety included total volume of blood loss within the first postoperative 24 h, the need for transfusion, the incidence of acute kidney injury, the mortality, the need for reoperation, and the length of stay in intensive care unit (ICU) and hospital. The efficiency was evaluated by total volume infusion, including colloid and crystalloids.

### Outcome

#### The total volume of infusion

The total volume of infusion was defined as the volume of albumin, HES, gelatin, and crystalloids used during surgery and the postoperative 24 h. In some studies, volumes were presented according to the type of fluid or several time intervals. The data were combined by the sum of the means for each interval and calculating the standard deviation of the sum of these data with a correlation coefficient of 0.5 between each time interval.

#### Blood loss

The total volume of blood loss was defined as the volume of blood loss during surgery and the first 24 h after surgery. The blood loss that was reported in articles was prioritized to be used because of its accuracy. If data in the required period was not reported, data of the maximum length of time was substituted for analysis. Some studies showed the data in a separate time interval. Therefore, we combined these adjacent time intervals by the sum of the means for each interval and calculating the standard deviation of the sum of these data with an assumed correlation coefficient of 0.5 between each space of time.

#### The frequency of transfusions

The transfusion requirements were accessed by the number of patients who received packed red blood cells (pRBCs) from the start of the surgery until 24 h. The percentages of patients receiving transfusion were calculated to the numbers of patients, which was rounded off to the integer. For these studies that presented the numbers of patients receiving blood transfusions in separate time intervals, we selected the nearest number of the total number of patients as the final data.

#### The number of days in ICU and hospital

This outcome represented the number of days for each group of patients in the ICU and hospital after the surgery. For some studies reported as the median and interquartile ranges, we assumed the data distribution was symmetrical and approximately normal. Therefore, the median values were used as the mean values, and the estimate of standard deviation was calculated as the interquartile ranges divided by 1.35. The same approach was applied to other outcomes.

#### Acute kidney injury, mortality, mortality and requirement for postoperative renal replacement therapy

The number of patients who suffered acute kidney injury (AKI) was calculated after the surgery without limiting any specific time window. The definition of AKI was consistent with the risk, injury, failure, loss and end-stage kidney (RIFLE) criteria [11]. For the mortality and need for RRT, the same approach was used to calculate. The mortality was recorded without limitation of time.

### Statistical analysis

We used RevMan software (Review Manager, version 5.2. the Nordic Cochrane Centre, Copenhagen, the Cochrane Collaboration, 2013) for data analysis. For continuous outcomes s, we used standardized mean difference (SMD). The inverse variance method was used to estimate study weights. We used the Mantel–Haenszel approach for dichotomous outcomes and reported a risk ratio (RR) with a corresponding 95% confidence interval (CI). Fixed-effects models were used to pool the weighted estimates among studies. Significant statistical heterogeneity was defined as P < 0.10 or I^2^ > 50%. A heterogeneity test was used to evaluate the heterogeneity of included studies.

## Results

Our research searched 6056 records. There were 1336 duplicates, 4411 records were excluded by screening the titles. One hundred sixty-three articles were not clinical trials. Fifty-six articles did not focus on studying albumin. Thirty-seven records were excluded for that they were not randomized controlled trials. And 30 records did not compare the albumin with HES. 13 records did not clear the type of HES or provide the data we needed. Our study ultimately included ten studies in our meta-analysis (Additional file 1: Figure S1), including a total.

The characteristics of selected studies were summarized in Table 1. Eight studies compared the albumin and 6% hydroxyethyl starch 130/0.4 in adults with cardiac surgery, and three studies were conducted in children or infants. Niemi et al. reported the hemodynamic changes of the same research of Schramko et al.

**Table 1:** The characteristics of included studies

Trials	Centers	Number of patients	Area	Gender M/F^a^	Age (years old)^a^	Type of surgery	Purpose
Niemi et al. (2008)	Single	30	Finland	9/6 vs. 11/4	61 (31–78) vs. 59 (34–73)^b^	On-pump cardiac surgery	Infusion fluid
Schramko et al. (2009)	Single	30	Finland	11/4 vs. 9/6	61 (48) vs. 59 (39) ^c^	Elective primary cardiac surgery	Infusion fluid
Choi et al. (2010)	Single	36	South Korea	6/12 vs. 5/13	55 ± 14 vs. 54 ± 12	Elective mitral valvular heart surgery with CPB	Priming solution
Cho et al. (2014)	Single	54	Korea	7/11 vs. 7/11	57 ± 17 vs. 64 ± 13	Complex cardiac surgery	Priming solution
Skhirtladze et al. (2014)	Single	240	Austria	53/23 vs. 52/29	66 (23–85) vs. 67 (28–87)^b^	Elective cardiovascular surgery with CPB	Infusion fluid
Maleki et al. (2016)	Single	60	Iran	21/9 vs. 17/13	66.07 ± 8.82 vs. 61.85 ± 9.10	Elective coronary artery bypass grafting surgery	Priming solutions
Duncan et al. (2020)	Single	141	America	47/22 vs. 44/28	71 (10)vs. 69 (9)^c^	Elective aortic valve replacement	Volume replacement
Hanart et al. (2009)	Single	119	Italy	38/21 vs. 32/28	0.91 (0.42–3.5) vs. 1.67 (0.67–3.83)^b^	Congenital heart disease necessitating CPB	Intraoperative fluid volume replacement
Van der Linden et al. (2013)	Two-center	61	Belgium and Austria	17/13 vs. 15/16	4.0 (2–9) vs. 5.2 (2–12)^b^	Elective cardiac surgery for congenital heart disease requiring the extracorporeal circulation device	Volume replacement
Patel et al. (2016)	Single	70	India	21/14 vs. 24/11	15.80 ± 13.11 vs. 16.20 ± 14.24	Cardiac surgery with CPB	Priming solution

*M/F* male/female, *CPB* cardiopulmonary bypass, of 1567 patients [12–21]

^a^HA vs. 6%HES 130/0.4; ^b^Interquartile range; ^c^Range

### Efficiency

#### The total volume of infusion

**Fig. 1:**
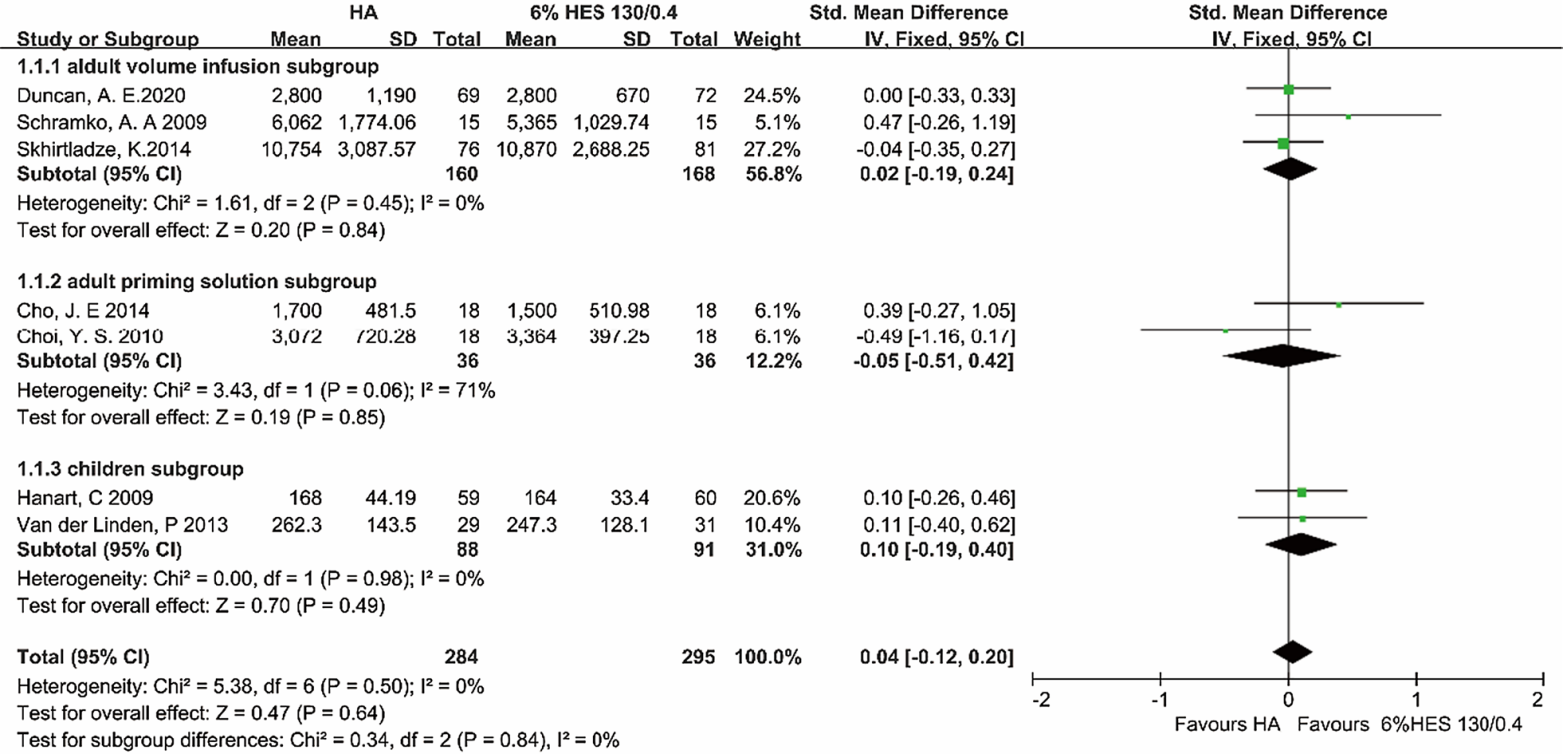
The total volume of infusion Unit of the total combined volume of infusions in the study of Hanart et al. and Van der Linden et al. was milliliters per kilogram body weight (ml/kg); other studies were milliliters (ml). *HA* human albumin, *HES* hydroxyethyl starch, *Std* standard difference, *CI* confidence interval. Fixed-effects models were applied to calculate a common effect

Seven trials (included 579 patients) reported the outcomes of total volume of infusion. There was no difference in total volume of infusion between compared groups [P = 0.84, I^2^ = 0%, P of I^2^ = 0.50; FEM: SMD = 0.04, 95% CI (− 0.12, 0.20)]. In adult volume infusion subgroup, the differences were not significant [P = 0.84, I^2^ = 0%, P of I^2^ = 0.45; FEM: SMD = 0.02, 95% CI (− 0.19, 0.24)]. In adult priming solution subgroup, the differences were not significant [P = 0.85, I^2^ = 71%, P of I^2^ = 0.06; FEM: SMD = − 0.05, 95% CI (− 0.51, 0.42)]. Comparing the two colloids in children subgroup, we found that it was also not significant [P = 0.49, I^2^ = 0%, P of I^2^ = 0.98; FEM: SMD = 0.04, 95% CI (− 0.12, 0.20)] (Fig. 1).

### Safety

#### Blood loss

**Fig. 2:**
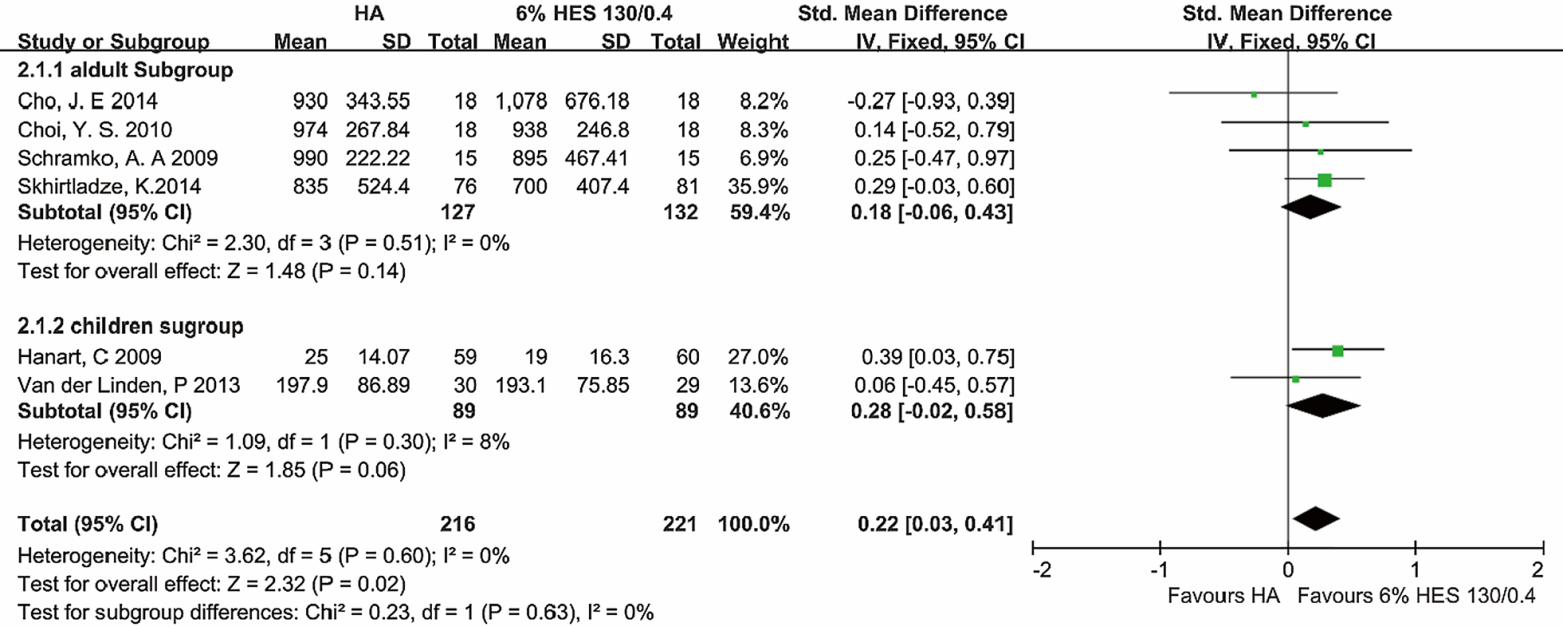
Blood loss. Units of the total combined volume of infusions were milliliters (ml). Units of Hanart et al. and Van der Linden et al. were milliliters per kilogram body weight (ml/kg). *HA* human albumin, *HES* hydroxyethyl starch, *Std* standard difference, *CI* confidence interval. Fixed-effects models were applied to calculate a common effect

Six trials were included to analyze the blood loss included total of 437 patients. A fixed effect model (FEM) analysis showed that the difference between albumin and hydroxyethyl starch 130/0.4 in blood loss [SMD: 0.22; 95% CI (0.03, 0.41)] was significant (p = 0.02), with no heterogeneity for the outcome (heterogeneity: P = 0.60, I^2^ = 0%). In adult subgroup, the differences were not significant [P = 0.14, I^2^ = 0%, P of I^2^ = 0.51; FEM: SMD = 0.18, 95% CI (− 0.06, 0.43)]. Comparing the two colloids in children subgroup, we found that it was significant [P = 0.06, I^2^ = 8%, P of I^2^ = 0.30; FEM: SMD = 0.28, 95% CI (− 0.02, 0.58)] (Fig. 2).

#### The frequency of transfusions

**Fig. 3:**
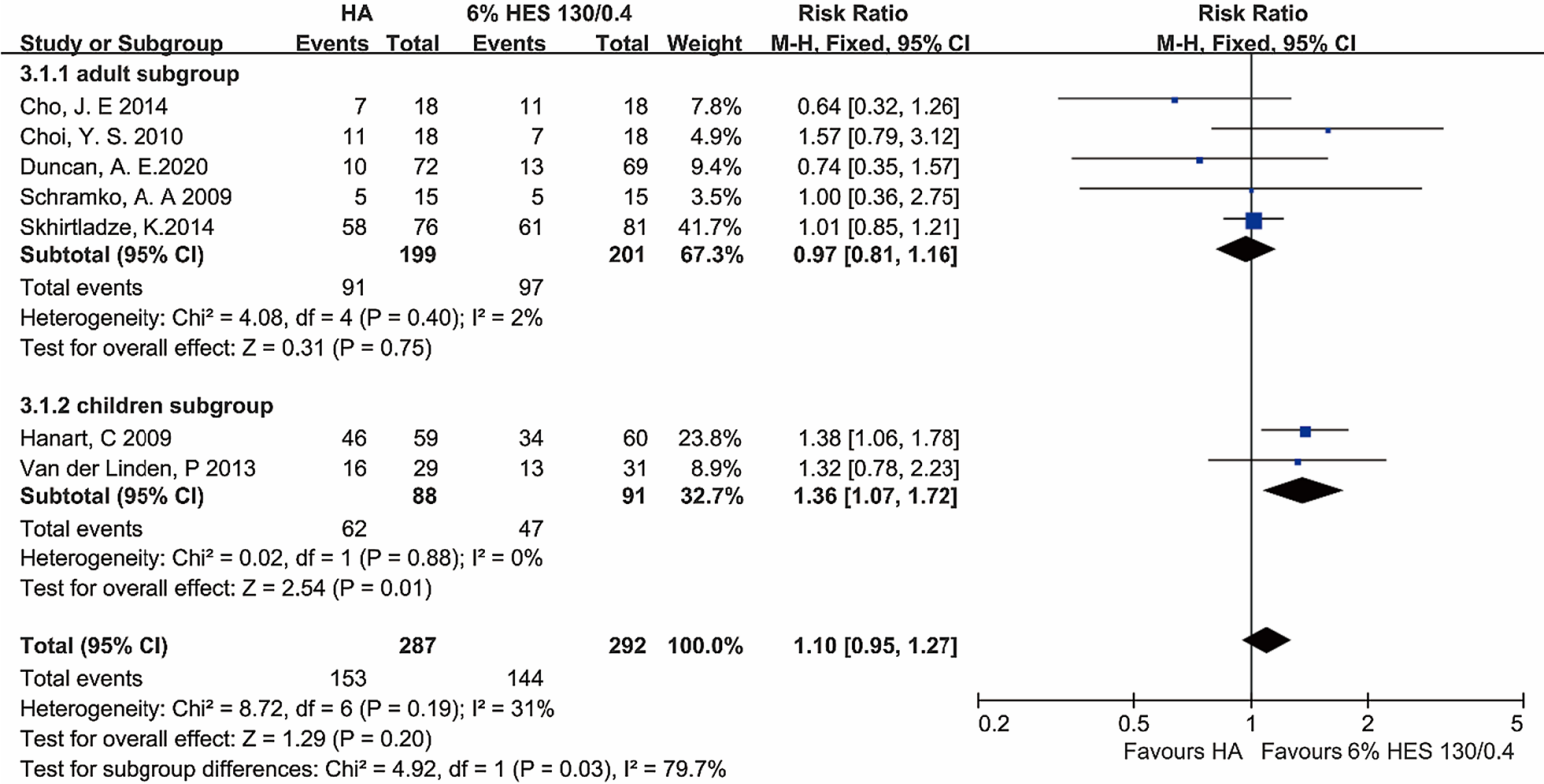
The frequency of transfusions. The numbers of Cho et al. and Choi et al., included the number of patients that need a transfusion in the first 24 h after the cardiac surgery. *HA* human albumin, *HES* hydroxyethyl starch, *CI* confidence interval; Fixed-effects models were applied to calculate a common effect

Seven trials (included 579 patients) contributed to the data of the frequency of transfusion. In the albumin group, 48% of patients (139 of 287) exposed to the pRBCs compared with 45% of patients (132 of 292) in the crystalloid group. There was no difference in the frequency of transfusions (P = 0.20, RR = 1.11; 95% CI (0.95, 1.27); I^2^ = 31%, P of I^2^ = 0.19) between the two groups with a fixed-effect model analysis. Low heterogeneity might account for the study of Hanart et al., which included younger children than other studies. None of the high-bias studies reported the frequency of transfusions outcomes. In adult subgroup, the differences were not significant [P = 0.75, I^2^ = 2%, P of I^2^ = 0.40; FEM: RR = 0.97, 95% CI (0.81, 1.16)]. However, comparing the two colloids in the children subgroup, we found that the difference was significant [P = 0.01, I^2^ = 0%, P of I^2^ = 0.30; FEM: RR = 1.36, 95% CI (1.07, 1.72)] (Fig. 3).

#### The days in ICU and hospital

**Fig. 4:**
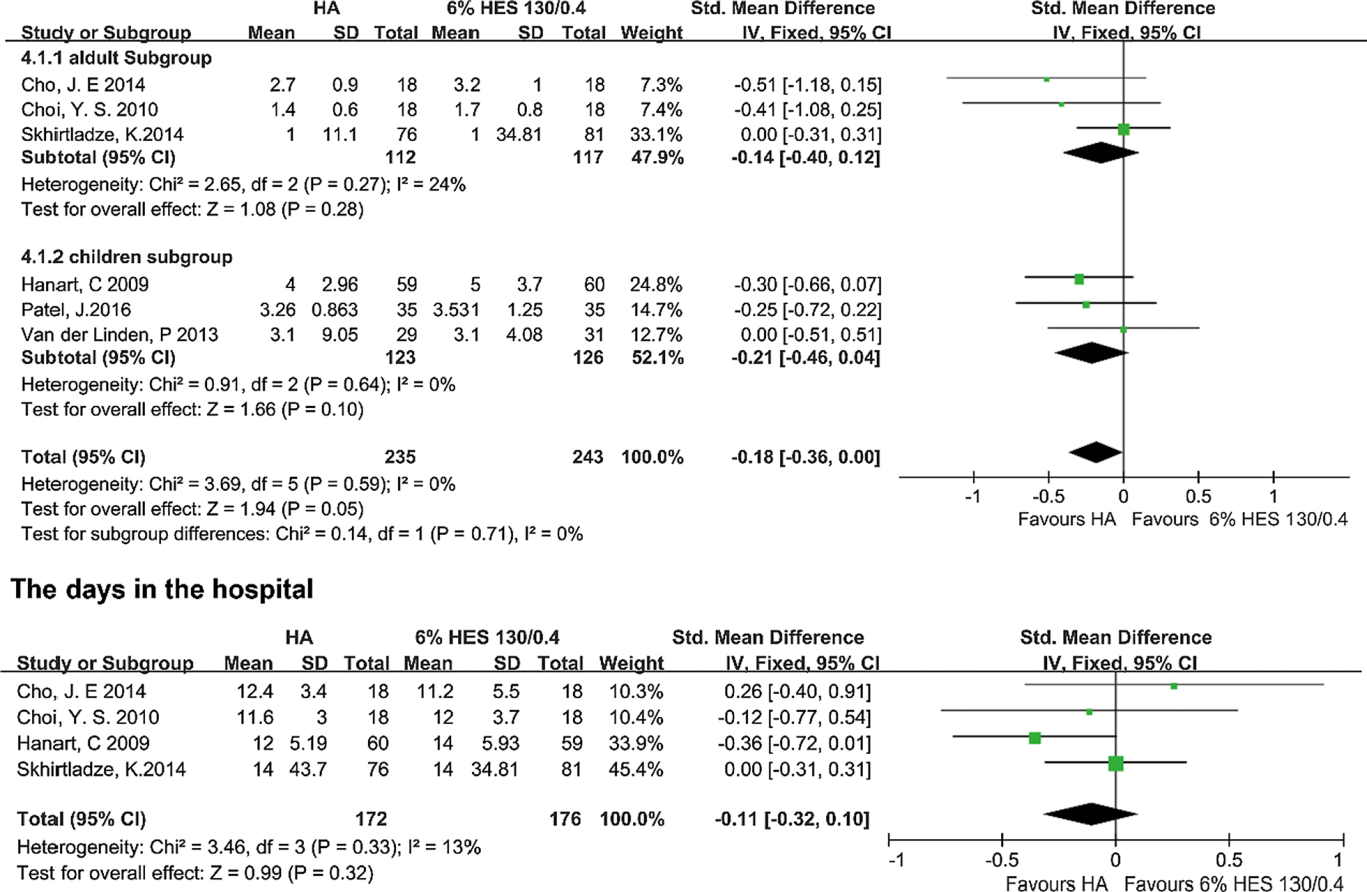
The days in ICU and hospital. *ICU* intensive care unit, *HA* human albumin, *HES* hydroxyethyl starch, *Std* standard difference, Fixed-effects models were applied to calculate a common effect. *CI* confidence interval

Six trials which included a total of 478 patients, showed the days in ICU. And five of them (348 patients in total) reported the data of days in the hospital. No difference was observed between the albumin group and hydroxyethyl starch 130/0.4 group of days in ICU [P = 0.05, I^2^ = 0%, P of I^2^ = 0.59; FEM: SMD = -0.18, 95% CI (-0.36, 0.00)]. For subgroup analysis, in adult subgroup, the differences were not significant [P = 0.28, I^2^ = 24%, P of I^2^ = 0.27; FEM: SMD = − 0.14, 95% CI (− 0.04, 0.12)]. However, comparing the two colloids in the children subgroup, we found that the difference was not significant [P = 0.10, I^2^ = 0%, P of I^2^ = 0.64; FEM: SMD = − 0.21, 95% CI (− 0.46, 0.04)] (Fig. 4).

**Fig. 5:**
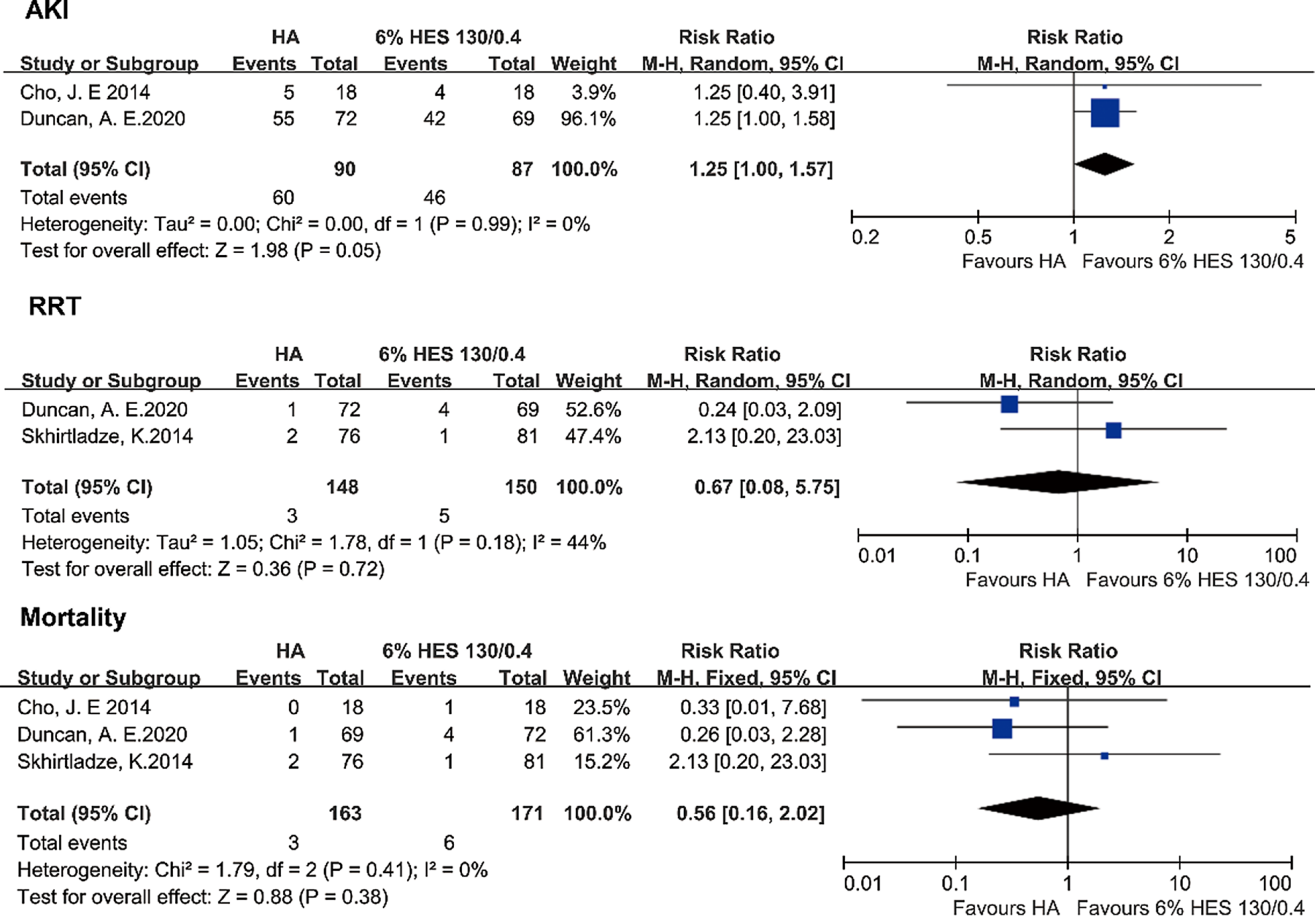
AKI, the requirement for postoperative renal replacement therapy and mortality. *AKI* acute kidney injury, *HA* human albumin, *HES* hydroxyethyl starch, *CI* confidence interval. Fixed-effects models were applied to calculate a common effect

The difference of the days in hospital was not significant neither [P = 0.32, I^2^ = 13%, P of I^2^ = 0.33; FEM: SMD = − 0.11, 95% CI (− 0.32, 0.10)] (Fig. 5).

### Acute kidney injury, the requirement for postoperative renal replacement therapy and mortality

Two trials that included 177 patients reported the incidence of AKI assessed by RIFLE classification. The difference was not significant between compared groups [P = 0.05, I^2^ = 0%, P of I^2^ = 0.99; FEM: RR = 1.25, 95% CI (1.00, 1.58)] (Fig. 5). Three trials (334 patients in total) reported the incidence of RRT. The analysis with the fixed-effect model demonstrated that the incidence of RRT was not significant in the two groups [P = 0.72, I^2^ = 44%, P of I^2^ = 0.18; FEM: RR = 0.67, 95% CI (0.08, 5.75)] (Fig. 5). The heterogeneity might account for that Duncan et al. reported the incidence of RRT 1 year after the surgery, while Skhirtladze et al. showed the postoperative data. Three trials reported the mortality of patients included 334 patients. No difference was observed in compared groups for the mortality [P = 0.46, I^2^ = 0%, P of I^2^ = 0.41; FEM: RR = 0.58, 95% CI (0.14, 2.43)] (Fig. 5).

### Risk of bias assessment

As shown in Additional file 1: Figure S2, the allocation concealment was not described in three trials [17, 20, 21]. The blinding of participants and personal was not described in one trial [13]. The blinding of outcome assessment was not described in four studies [14, 17, 19, 20]. The blinding of outcome assessment was not used in one trial [16]. The study of Patel et al. reported incomplete data and selective reported some of the results, such as the data of blood loss, blood and blood product requirements, which were negative results, were not reported in the articles[21]. No other bias were reported in included trials.

### Sensitivity analysis

We conducted a sensitivity analysis on the index of blood loss by studying the influence of a single study on the total combined effect. The study found that if any of the studies were removed, the heterogeneity of blood loss for all studies was I^2^ = 0%. In the subgroup analysis, excluding one of Choi et al. and Schramko et al. showed that the heterogeneity of I^2^ = 12% in the adult group. Because the study of Skhirtladze et al. and Hanart et al. has a significant weight in the meta-analysis. The removal of any of these two studies resulted in statistically significant changes (P = 0.12).

### Publication bias

Funnel plots were used to evaluate the publication bias of the included studies, and the publication bias of blood loss was added to the indicators in Additional file 1: Figure S3. According to the funnel plots, The studies in the funnel chart are symmetrical, indicating that the publication bias is slight.

## Discussion

The main finding of this study shows the efficacy of HA, which was assessed by the total volume of infusion, was not superior to 6% HES (130/0.4). Concerning the safety of the two fluids, the HA presented more blood loss risk than 6% HES (130/0.4). The frequency of transfusion in patients undergoing cardiac surgery was comparable in HA and the 6% HES (130/0.4) group. For children with cardiac surgery, HES 130/0.4 showed a reduced frequency of transfusion compared with HA. No differences were found in the days in ICU and hospital, the incidence of AKI and the requirements for RRT and mortality between the two groups. Thus, our analysis indicated that HA and 6% HES (130/0.4) might have similar effects in the fluid administration for patients with cardiac surgery.

To identify the efficiency of HES 130/0.4, we assessed the total volume of infusion in patients with cardiac surgery. The comprehensive consideration decided the need for infusion for the multiple factors such as cardiac preload and fluid balance parameters. Jacob et al. suggested that individual differences for the total volume of infusion might regress toward the mean, which was more reliable because this data combined the multiple parameters [22]. This study showed no difference in the total volume of infusion between compared groups, which was consistent with previous studies [22]. Similar efficiency provides a possibility for HES 130/0.4 as a substitute for albumin, but the more reliable conclusion for their comparison of efficiency needs a large multiple center trial.

The frequency of transfusion of pRBCs was related to the long-term survival after cardiac surgery [23]. Consequently, the blood loss and transfusion of pRBCs were crucial indicators to access the safety of fluids for volume replacement in patients undergoing cardiac procedures. The observational study found that 6% HES (130/0.4) increased transfusions because of its greater hemodilution, clotting disturbances, and bleeding in adult cardiac surgery patients [24]. However, other studies found 6% HES (130/0.4) did not increase postoperative bleeding [25]. Thus, the results of trials that evaluated the safety of 6% HES (130/0.4) in cardiac surgical patients were inconsistent. Similar to a previous meta-analysis [22], this study found the blood loss with HA was significantly less than that with HES 130/0.4. However, the difference was negligible in the adult and children subgroup with cardiac surgery. However, there was no significant difference in the transfusion requirement between groups. The adult subgroup analysis also showed similar results. In the children subgroup, the transfusion requirements with HA were higher when compared with HES 130/0.4, but this subgroup only included a small number of patients, which might be influenced by included study with large sample size. Nevertheless, these results differed from the latest meta-analysis [22], which might be because the number of trials they included in the study was limited. The estimation methods of the standard deviation were a little different. In conclusion, 6% HES (130/0.4) might be a safe alternative to HA in pediatric heart surgery, which was similar to a previous study in children [26]. However, more trials with larger samples were still needed to confirm these results.

The ICU and hospital stays were also evaluated. Jacob and his colleagues showed that the HA group had shorter ICU stays than the HES130/0.4 group [22]. Moreover, a previous study showed that HES 130/0.4 was associated with increased hospital length of stay [27]. However, the days in ICU and hospital were similar in two groups regardless of adult and children in this study. The difference in the ICU stays might be that more studies and a larger sample size were included in this study.

It should be noted that numerous non-medical factors would, such as the patients’ requirements, the healing of the wound or weekend discharges, could influence the length of hospital stays. These might be one of the reasons for the heterogeneity. Hence, choosing the days in the hospital as the outcome must be cautious.

AKI is the major concern with HES use in critically ill patients, particularly in patients with sepsis [28, 29]. But in patients with cardiac surgery, it’s debatable whether 6% HES (130/0.4) was associated with AKI. Compared with crystalloid or gelatin, 6% HES (130/0.4) increased the incidence of AKI and the risk of RRT in cardiac surgery, especially in the early postoperative period [25, 27, 30]. While recent studies found that 6% HES (130/0.4) did not increase the incidence of postoperative AKI in cardiac surgery [29–31]. The three selected studies that considered AKI or RRT as an outcome were conducted in adults. Cho et al. used HES as a priming fluid, while other studies used HES as fluid resuscitation. These might affect the renal outcome. Therefore, more studies are needed to clarify its effect on renal function. Cho et al. found similar results between two groups for the incidence of AKI, RRT, mortality, which was consistent with previous studies [22]. Furthermore, Duncan et al. analyzed the urinary concentration of NGAL and IL-18, two early predictive biomarkers of AKI, in cardiac patients. They found the changes of urinary, renal biomarkers in the HES 130/0.4 group were comparable to HA. The researcher thought that the study was limited to their small sample size, which cannot make a firm conclusion for the renal function [18]. Furthermore, Skhirtladze et al. did not clearly define the time for postoperative mortality and did not have a clear definition of time for AKI [15]. Therefore, the effect of HES on AKI and mortality still needs to be further explored in clinical studies with the renal outcome.

On the other hand, HES is associated with higher AKI incidence in septic patients [28, 29]. Unfortunately, these studies did not include data related to sepsis complications. After reviewing the literature, due to the limited number of studies that reported AKI-related data in patients with cardiac surgery, the effect of sepsis complications on HES related AKI is not apparent at present. In the future, septic complications are noticeable factors that need to be considered in the study of HES and AKI in cardiac surgery.

The limitations of this study were as following: Firstly, most of the included studies were single-center studies with a small sample size worldwide, which might increase the heterogeneity of this study. Besides, some outcomes were missing in some studies. The large sample trials have a relatively high effect on our results. Furthermore, the low numbers of total participants make this meta-analysis underpowered for the outcomes of mortality, renal dysfunction.

## Conclusion

In conclusion, HA and HES130/0.4 had an equivalent effect on the volume expansion. But HES130/0.4 showed less blood loss than HA in cardiac surgery. The HA and HES130/0.4 were comparable for other safety parameters, such as the frequency of transfusions, the number of days in ICU and hospital, AKI, RRT and mortality. Hence, this study provided evidence for the HES130/0.4, which might substitute HA in patients with cardiac surgery. However, the safety of HES130/0.4 for renal outcomes still needed to be validated by more trials.

## Supplementary Information

**Additional file 1: Figure S1**. Process of randomized trial selection. **Figure S2**. Risk of bias summary Niemi, T. et al. reported the hemodynamic changes of the same study of Schramko, A. A. et al. **Figure S3**. The funnel plots for the outcome of blood loss. Std Standard difference, SMD, Std mean difference. SE, standard error. **Table S1**. PICOS. **Table S2**. The summary of the results.

## Data Availability

All data relevant to the study are included in the article or uploaded as supplementary information.
