# Supplementary material for: The comparison of albumin and 6% hydroxyethyl starches (130/0.4) in cardiac surgery: a meta-analysis of randomized controlled clinical trials
Source: BMC Surg. 2021 Sep 11;21:342. doi: 10.1186/s12893-021-01340-x (PMC8436511; doi:10.1186/s12893-021-01340-x)
Supplement: Supplementary file 1 — Additional file 1: Figure S1. Process of randomized trial selection. Figure S2. Risk of bias summary Niemi, T. et al. reported the hemodynamic changes of the same study of Schramko, A. A. et al. Figure S3. The funnel plots for the outcome of blood loss. Std Standard difference, SMD, Std mean difference. SE, standard error. Table S1. PICOS. Table S2. The summary of the results. [file 12893_2021_1340_MOESM1_ESM.docx]

**The additional material**

**Title: The comparison of albumin and 6% hydroxyethyl starches (130/0.4) in cardiac surgery: a meta-analysis of randomized controlled clinical trials**

**Ling Wei^1^, Dongping Li^2^, Lin Sun^1^**

1 Department of Nephrology, The Second Xiangya Hospital, Central South University, Changsha, China

2 Department of Cardiothoracic Surgery, The Second Xiangya Hospital, Central South University, Changsha, China

*** Correspondence:**Lin Sun, MD.Ph.D

Department of Nephrology

Second Xiangya Hospital/Central South University

Changsha, China

Tel: 86-0731-85292064

E-mail: sunlin@csu.edu.cn

**Short title:** albumin and 6% hydroxyethyl starches (130/0.4) in cardiac surgery

**Keywords:** albumin, hydroxyethyl starches, cardiac surgery, randomized controlled clinical trials, meta-analysis

**Figure S1.** Process of randomized trial selection.


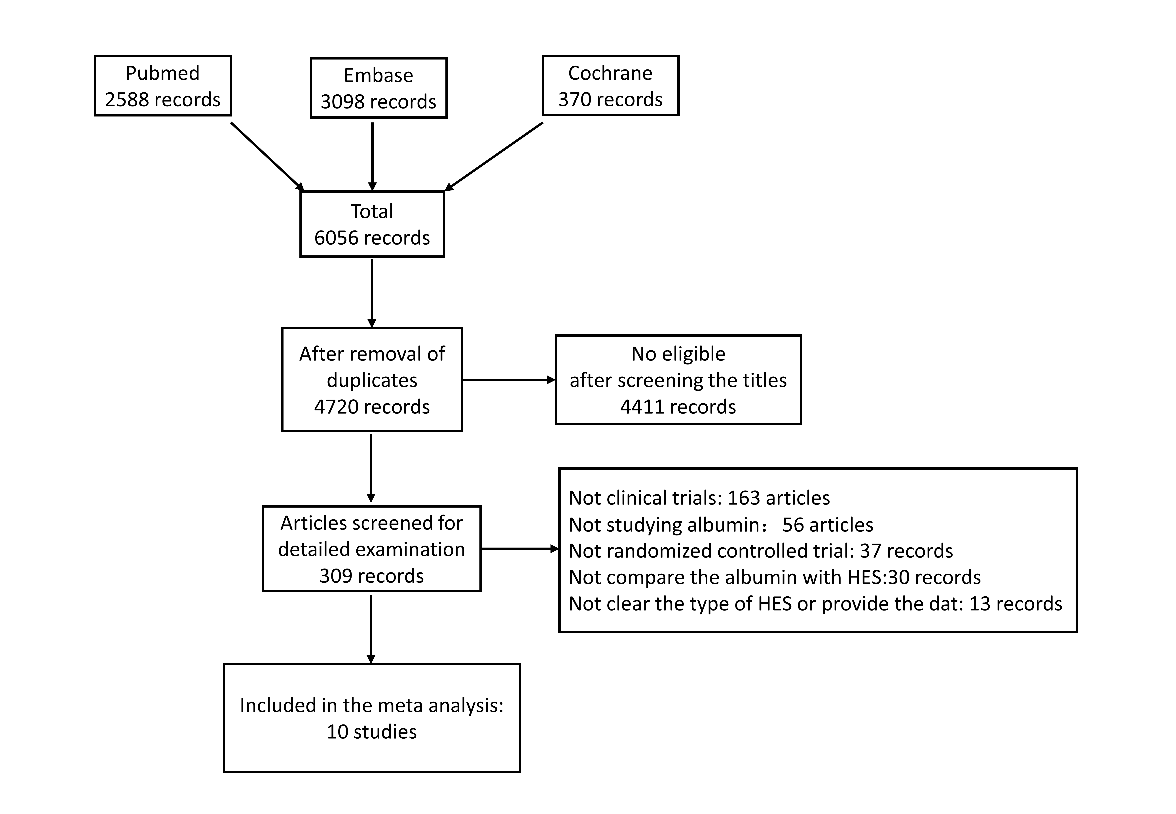


**Figure S2** Risk of bias summary Niemi, T. et al. reported the hemodynamic changes of the same study of Schramko, A. A. et al.


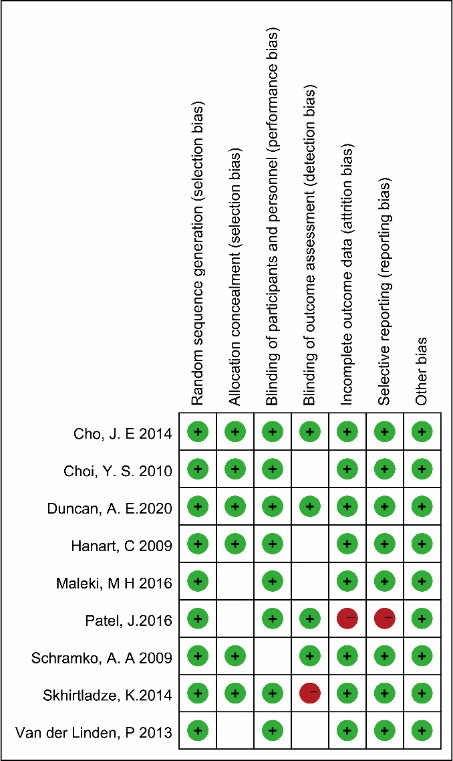


**Figure S3 The funnel plots for the outcome of blood loss** Std Standard difference, SMD, Std mean difference. SE, standard error


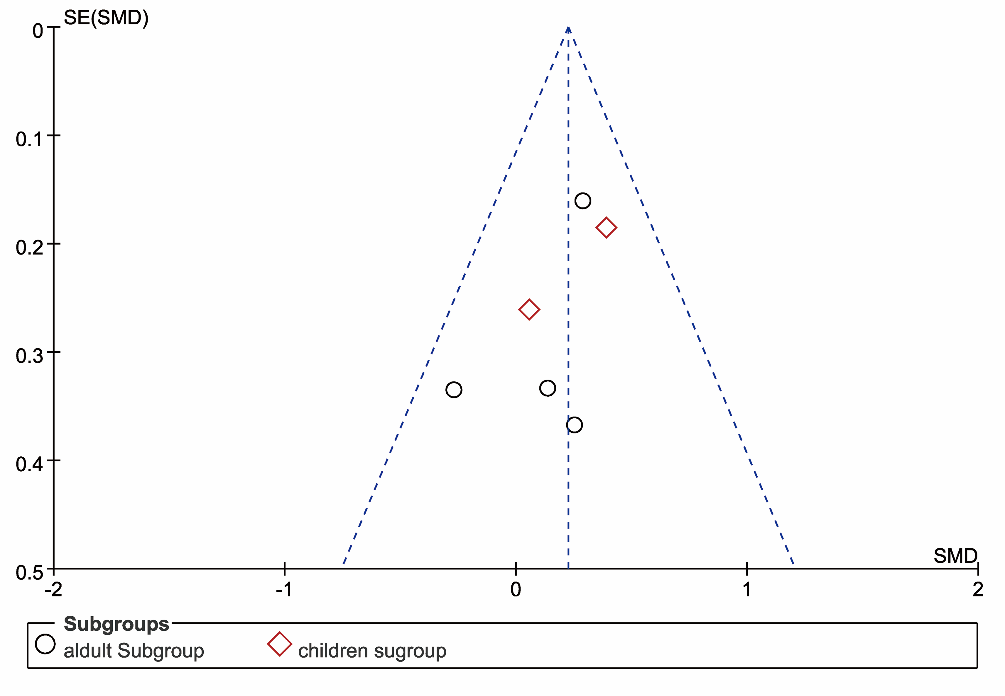


**Table S1 PICOS**

| Participants | the patients had cardiac surgery |
| --- | --- |
| Intervention | Use hydroxyethyl starches (130/0.4) as priming solution or volume replacement |
| Comparison | Use albumin as a priming solution or volume replacement |
| Outcome | (1) total volume infusion, (2)total volume of blood loss within first postoperative 24 hours, (3) the need for transfusion, the incidence of acute kidney injury, RRT and mortality, (5) the need for reoperation, (6) the length of stay in intensive care unit (ICU) and hospital |
| Study design | randomized controlled clinical trials |

**Table S2 The summary of the results**

| Outcome | Total | | | | |  | Adult | | | |  | Children | | | | |
| --- | --- | --- | --- | --- | --- | --- | --- | --- | --- | --- | --- | --- | --- | --- | --- | --- |
|  | Patients | p | SMD/RR | 95%CI | I^2^ (%) |  | p | SMD/RR | 95%CI | I^2^ (%) |  | p | SMD/RR | 95%CI | I^2^ (%) |  |
| Total volume of infusion | 579 | 0.84 | 0.04 | (-0.12, 0.20) | 0 |  | 0.92 | 0.01 | (-0.19, 0.21) | 22 |  | 0.49 | 0.04 | (-0.12, 0.20) | 0 |  |
| Blood loss | 437 | 0.02 | 0.22 | (0.03, 0.41) | 0 |  | 0.14 | 0.18 | (-0.06, 0.43) | 0 |  | 0.06 | 0.28 | (-0.02, 0.58) | 8 |  |
| Frequency of transfusions | 579 | 0.2 | 1.11 | (0.95, 1.27) | 31 |  | 0.75 | 0.97 | (0.81, 1.16) | 2 |  | 0.01 | 1.36 | (1.07, 1.72) | 0 |  |
| days in ICU | 478 | 0.05 | -0.18 | (-0.36, 0.00) | 0 |  | 0.28 | -0.14 | (-0.04, 0.12) | 24 |  | 0.1 | -0.21 | (-0.46, 0.04) | 0 |  |
| days in hospital | 348 | 0.32 | -0.11 | (-0.32, 0.10) | 13 |  | / | / | / | / |  | / | / | / | / |  |
| AKI | 177 | 0.05 | 1.25 | (1.00, 1.58) | 0 |  | / | / | / | / |  | / | / | / | / |  |
| RRT | 298 | 0.72 | 0.67 | (0.08, 5.75) | 44 |  | / | / | / | / |  | / | / | / | / |  |
| mortality | 334 | 0.46 | 0.58 | (0.14, 2.43) | 0 |  | / | / | / | / |  | / | / | / | / |  |

Std Standard difference, SMD, Std mean difference, RR, risk ratio，

The additional material

1. The retrieval strategy
2. **Cochrane**

#1 MeSH descriptor: [Albumins] this term only

#2 (Albumin*):ti,ab,kw

#3 #1 or #2

#4 MeSH descriptor: [Cardiac Surgical Procedures] 1 tree(s) exploded

#5 ((“cardiac surgery” or “cardiovascular surgery” or “heart surgery” or “coronary surgery” or “coronary artery bypass grafting” or “valve surgery” or “valve replacement” or “valve repair” or “aortic surgery” or “coronary artery bypass” or “ventricular assist device” or “cardiac transplantation” or “cardiopulmonary bypass”)):ti,ab,kw

#6 #4 or #5

#7 #3 and #6

1. **Embase**

#1. 'albumins'/exp OR albumins

#2. albumin:ab,ti

#3. #1 OR #2

#4. 'cardiac surgical procedures'/exp OR 'cardiac surgical procedures'

#5. 'cardiac surgery':ab,ti OR 'cardiovascular surgery':ab,ti OR 'heart surgery':ab,ti OR 'coronary surgery':ab,ti OR 'coronary artery bypass grafting':ab,ti OR 'valve surgery':ab,ti OR 'valve replacement':ab,ti OR 'valve repair':ab,ti OR 'aortic surgery':ab,ti OR 'coronary artery bypass':ab,ti OR 'ventricular assist device':ab,ti OR 'cardiac transplantation':ab,ti OR 'cardiopulmonary bypass':ab,ti

#6. #4 OR #5

#7. #3 AND #6

1. **Pubmed**

#1 (albumins[MeSH Terms]) OR (albumin*[Title/Abstract])

#2 Cardiac Surgical Procedures[MeSH Terms]

#3 "cardiac surgery"[Title/Abstract] OR "cardiovascular surgery"[Title/Abstract] OR "heart surgery"[Title/Abstract] OR "coronary surgery"[Title/Abstract] OR "coronary artery bypass grafting"[Title/Abstract] OR "valve surgery"[Title/Abstract] OR "valve replacement"[Title/Abstract] OR "valve repair"[Title/Abstract] OR "aortic surgery"[Title/Abstract] OR "coronary artery bypass"[Title/Abstract] OR "ventricular assist device"[Title/Abstract] OR "cardiac transplantation"[Title/Abstract] OR "cardiopulmonary bypass"[All Fields]

#4 (#2) OR (#3)

#5 (#1) AND (#4)
